# Heterologous production of human papillomavirus type-16 L1 protein by a lactic acid bacterium

**DOI:** 10.1186/1756-0500-2-167

**Published:** 2009-08-24

**Authors:** Naima G Cortes-Perez, Pascale Kharrat, Philippe Langella, Luis G Bermúdez-Humarán

**Affiliations:** 1 Equipe Interactions des bactéries commensales et probiotiques avec l'hôte, Unité d'Ecologie et physiologie du Système Digestif, Institut National de la Recherche Agronomique, 78352 Jouy-en-Josas, France

## Abstract

**Background:**

The expression of vaccine antigens in lactic acid bacteria (LAB) is a safe and cost-effective alternative to traditional expression systems. In this study, we investigated i) the expression of Human papillomavirus type 16 (HPV-16) L1 major capsid protein in the model LAB *Lactococcus lactis *and ii) the ability of the resulting recombinant strain to produce either capsomer-or virus-like particles (VLPs).

**Results and conclusion:**

HPV-16 L1 gene was cloned into two vectors, pCYT and pSEC, designed for controlled intra- or extracellular heterologous expression in *L. lactis*, respectively. The capacity of *L. lactis *harboring either pCYT:L1 or pSEC:L1 plasmid to accumulate L1 in the cytoplasm and supernatant samples was confirmed by Western blot assays. Electron microscopy analysis suggests that, L1 protein produced by recombinant lactococci can self-assemble into structures morphologically similar to VLPs intracellularly. The presence of conformational epitopes on the *L. lactis*-derived VLPs was confirmed by ELISA using an anti-HPV16 L1 capsid antigen antibody. Our results support the feasibility of using recombinant food-grade LAB, such as *L. lactis*, for the production of L1-based VLPs and open the possibility for the development of a new safe mucosal vector for HPV-16 prophylactic vaccination.

## Background

Human papillomavirus type 16 (HPV-16) infection is closely associated with the development of cervical cancer (CxCa) [1], the second cause of cancer-related deaths in women worldwide (~250 000 annually) [2]. Therefore, a prophylactic vaccine against HPV-16 is thus a priority to prevent this type of cancer. HPV-16 L1 major capsid protein is able to self-assemble into virus-like particles (VLPs) which are structures that are morphologically similar and immunogenic as to native HPV [17]. Prophylactic vaccines based on highly purified VLPs were successfully used in trials in women with a significant reduction observed in the incidence of both HPV-16 infection and HPV-16 related CxCa [3], and now two vaccines, Gardasil and Cervarix, have been approved for use against this cancer. In developing countries, where about 80% of CxCa occurs [2], immunization programs would be more efficient and economical if vaccines are temperature stable, required less doses to immunize and could be administered without the need for specially trained personnel and instruments (*eg*. needles).

Alternatively, mucosal vaccines (*e.g*. administered by oral, intranasal, rectal or vaginal route) are more convenient than systemic vaccines, because they are easier to administer, relatively cheap to produce and less invasive, an especially important criteria when used with children and immunosuppressed patients [5]. Furthermore, mucosal vaccines can stimulate serum-IgG and mucosal-IgA Abs (to neutralize toxins and viruses) and induce CTL activities [6].

Lactic acid bacteria (LAB) are non-pathogenic and non-invasive Gram-positive bacteria considered as being promising candidates for controlled and targeted administration of vaccine antigens to the mucosal immune system [7-9]. Because of their long and safe association with humans and their food such bacterial vectors represent a good alternative to the use of classical attenuated pathogenic bacterial carriers. Moreover, some LAB strains are well known for their probiotic effect in humans [for a review see Reference 9]. Previously, we reported HPV-16 E7 production in *Lactococcus lactis*, the model LAB [10-12]. Mucosal immunization with recombinant *L. lactis *expressing E7 antigen and secreting biologically active IL-12 induced an E7-specific response and displayed therapeutic effects against HPV-16-induced tumors in mice [11,13]. This was the first study demonstrating the potential of recombinant non-pathogenic and non-invasive *L. lactis*-based vaccines to help control HPV-related CxCa. An alternative strategy to combat HPV-related cancer is the prevention of infection with HPV. VLPs obtained by the expression of HPV L1 major virion capsid protein using recombinant vectors are good candidates for a prophylactic HPV vaccine. Expression of HPV-16 L1 protein (for vaccine purposes) via heterologous LAB, including *Lactobacillus casei *and *L. lactis*, has been previously shown to generate VLPs [7,14,15]. Therefore, in this study, we sought to determine whether *L. lactis *is able to produce HPV-16 L1 protein using the NICE system [32] and whether L1 protein produced by recombinant *L. lactis *is able to self-assemble into either capsomers or VLPs.

## Results and discussion

### HPV-16 L1 production by *Lactococcus lactis*

**Figure 1:**
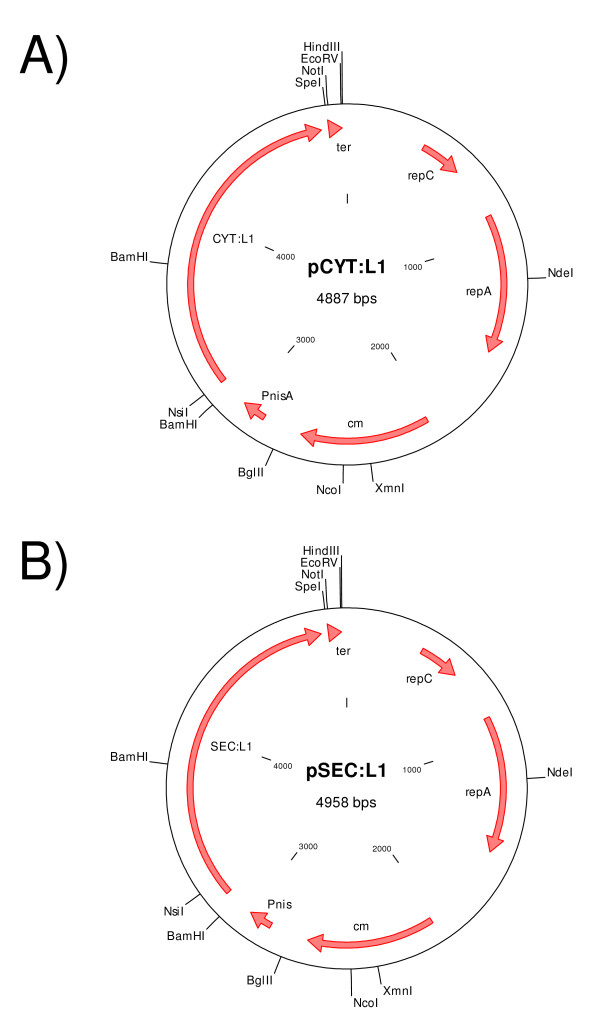
**Schematic representation of pCYT:L1 (A) and pSEC:L1 (B) plasmids**. A DNA fragment encoding L1 (see Materials and methods) was fused in frame with a DNA fragment containing the Usp45 signal peptide (pSEC:L1), derived from the predominant *L. lactis*-secreted protein. In pCYT:L1, the fragment encoding SP_Usp45 _is absent. In these plasmids, L1 expression is controlled by the nisin-inducible promoter (P_*nisA*_) and harbors the Usp45 ribosome binding site and the *rho*-independent *trpA *transcription terminator (ter) [10] for clone stability.

**Figure 2:**
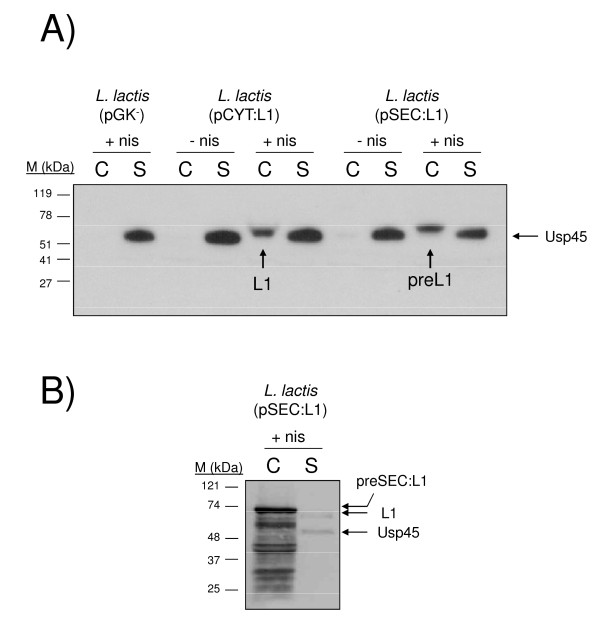
**Expression of HPV-16 L1 protein by recombinant *L. lactis***. A) *L. lactis *strains were grown and induced with 1 ng/ml of nisin for 1 h. After centrifugation, non-induced (- nis) and induced (+ nis) culture samples were treated as described in materials and methods and L1 production and secretion analyzed by Western blot. *L. lactis *strain contains pCYT:L1 plasmid encoding for a cytoplasmic form of L1 and *L. lactis *strain contains pSEC:L1 encoding for the precursor preL1 (*i*.*e*. SP_Usp45 _fused to L1). B) *L. lactis *(pSEC:L1) strain was grown and induced with 10 ng/ml of nisin for 1 h. After centrifugation, induced (+ nis) culture samples were treated as described in results and discussion and L1 production and secretion analyzed by Western blot. Arrows indicate positions of L1 mature and precursor forms. Abbreviations: C, cell lysates; S, supernatant fraction; M, positions and sizes of molecular mass markers.

The capacity of *L. lactis *to produce and secrete L1 protein was examined using lactococci strains harbouring pCYT:L1 and pSEC:L1 plasmids (Figure 1), respectively. Non-induced and induced culture samples were examined by Western blot using HPV-16 L1-specific monoclonal antibodies. As shown in Figure 2A, no L1 signal was detected in either cell or supernatant fractions of induced cultures of the negative control *L. lactis *(pGK^-^). In the absence of nisin, no L1 signal was detected in either *L. lactis *(pCYT:L1) or *L. lactis *(pSEC:L1) strain, indicating that NICE system allows tight control of gene expression. Induced cultures of *L. lactis *(pCYT:L1) strain resulted in a clear band at the expected size for native L1 (~58 kDa) was observed in the cell fraction whereas no signal was detected in the supernatant. Similar analysis of *L. lactis *(pSEC:L1) resulted in a clear band in the cell fraction corresponding to SP_Usp45_-L1 precursor (pre-L1, ~60 kDa). Unfortunately, since a major non-specific band (which migrates at the expected size for native L1: ~58 kDa) reacts with anti-L1 antibodies in supernatant samples, L1 secretion cannot be determined. This non-specific band most probably corresponds to Usp45, the predominant *L. lactis*-secreted protein, and frequently detected by immunoblotting when using protein G-horseradish peroxidase conjugate [10,16].

In order to better determine the secretion of L1 protein by recombinant *L. lactis *(pSEC:L1) strain, we modified the protocol of immunoblotting by using a higher dilution of protein G-horseradish peroxidase conjugate (1:10,000) and a higher concentration of HPV-16 L1-specific monoclonal antibody (0.5 μg/ml). We also used a higher dose of nisin (*ie*. 10 ng/ml) for the induction of L1 expression. As shown in Figure 2B, the modifications in the protocol of immunoblotting and induction resulted in two bands, a clear band in the cell fraction corresponding to SP_Usp45_-L1 precursor (pre-L1, ~60 kDa), and a weak band in the supernatant fraction corresponding to the secreted mature L1 protein. Smaller-sized L1 products are also observed, reflecting degradation by HtrA or ClpP proteases or incomplete L1 synthesis at high expression levels (*ie*. 10 ng/ml of nisin). L1 secretion appears to be low, as about only 5% of the protein is detected in the supernatant.

### Production and secretion of HPV-16 L1 virus-like particles by *L. lactis*

**Figure 3:**
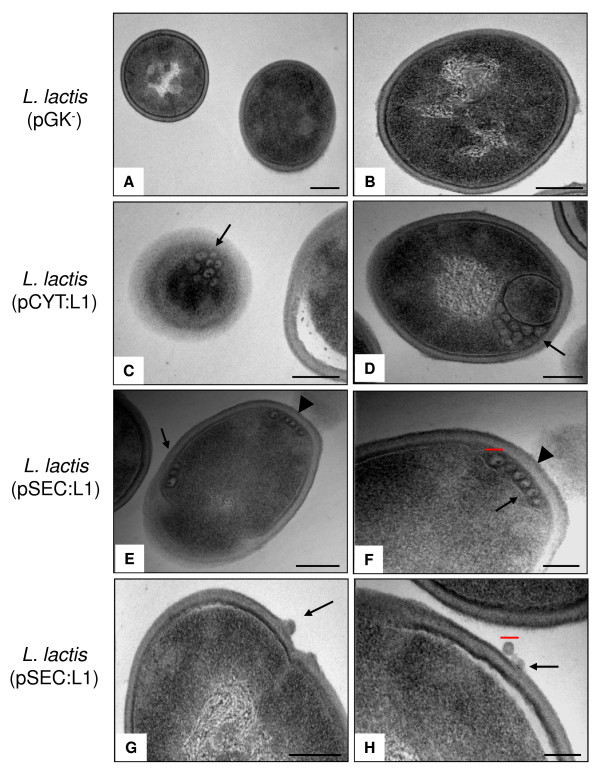
**Production and secretion of VLPs by *L. lactis *strains**. Electron micrographs of *L. lactis *(pGK^-^) (panel A and B), *L. lactis *(pCYT:L1) (panel C and D) and *L. lactis *(pSEC:L1) (panel E to H). The different strains were grown and prepared as described in material and methods. *L. lactis*-derived VLPs (arrows) have diameters ranging from ~30 to 50 nm (red bars = 50 nm). In *L. lactis *(pSEC:L1) strain, VLPs are localized at the intracellular periphery of the bacteria cells (arrowheads). Bars = 100 nm. Magnifications, A X50,000; B-G: X85,000; H: X140,000.

To determine whether recombinant L1 protein undergoes intracellular assembly into either capsomers or VLPs, TEM analysis was carried out. As shown in Figure 3 (panel C and D), structures morphologically similar to VLPs are present in *L. lactis *(pCYT:L1) strain and have similar sizes (30–50 nm in diameter) and morphologies as VLPs produced in other prokaryotic and eukaryotic systems (~55 nm) [14,15,17-20]. Surprisingly, VLPs are also present in the cytoplasmic fraction of *L. lactis *(pSEC:L1) strain (Figure 3, panel E and F); however, most of these VLPs are localized at the intracellular periphery of the bacterial cells (arrowheads). This phenomenon could be due to the self-assembly of small quantities of SP_Usp45_-L1 precursor into VLPs, which are dealt with the *L. lactis *secretion machinery via the translocon complex (early steps in the secretion) [21], suggesting that the Usp45 signal peptide (27 amino acids residues) does not interfere with intracellular assemblage of VLPs. Interestingly, some of these VLPs appear to be secreted or released out into the external medium (arrows) (Figure 3, panel G and H) by an unknown mechanism. It may be possible that the nascent secreted L1 protein undergoes self-assemble into VLPs during later stages of the secretion process. No VLPs were observed in negative control *L. lactis *(pGK^-^) (Figure 3, panel A and B).

### *L. lactis*-produced VLPs are recognized by a conformational antibody

**Figure 4:**
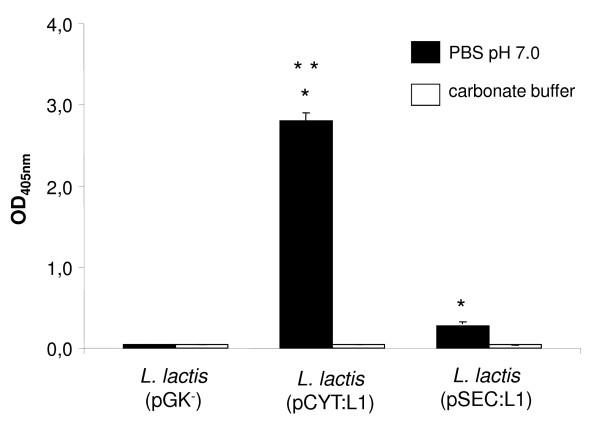
***L. lactis*-derived VLPs display conformational epitopes**. ELISA plates were coated with total protein cell extracts (diluted in PBS pH 7.0) obtained from either *L. lactis *strain and assayed with a conformational anti-HPV16 L1 antibody. The data are the means of two independent experiments. Bars represent the means ± standard deviations. Statistically significant differences (P < 0.05) obtained with JMP software are denoted by an asterisk (*) between recombinant *L. lactis *strains and control *L. lactis *(pGK-) strain or by two asterisks (**) between *L. lactis *(pCYT:L1) and *L. lactis *(pSEC:L1) strain.

In order to determine whether conformational epitopes are retained and displayed on the *L. lactis*-produced VLPs, an ELISA assay was performed using a monoclonal antibody that recognizes conformational epitopes present in HPV-16 VLPs (see material and methods). Total protein cell extracts from *L. lactis *strains were assayed using a conformational anti-HPV16 L1 antibody. As shown in Figure 4, cell extracts from *L. lactis *(pCYT:L1) and *L. lactis *(pSEC:L1) strains resuspended in PBS pH 7.0 (assembled VLPs) react with the anti-HPV16 L1 capsid antibody, indicating that conformational epitopes are displayed on *L. lactis*-derived VLPs. Note that the *L. lactis *(pCYT:L1) ELISA signal is significantly higher with *L. lactis *(pSEC:L1) strain. This could be explained in part by a higher quantity of intracellular-VLPs in *L. lactis *(pCYT:L1) strain, as observed by TEM analysis (Figure 3, panel C and D). Indeed, ELISA results are based on cell lysates to coat the plates and *L. lactis *(pSEC:L1) strain was designed for secretion. Hence, it does not retain much VLPS and/or L1 protein intracellularly and most VLPs are secreted to the external medium (Figure 3, panel G and H).

Samples from the negative control *L. lactis *(pGK^-^) do not react with the conformational antibody (Figure 4). As expected, the anti-HPV16 L1 capsid antibody was not able to recognize protein cell extracts from either *L. lactis *strain resuspended in carbonate buffer pH 10.0 (disassembled VLPs, data not shown).

## Conclusion

Infections with HPV are closely related to the development of CxCa [1]. For this reason, several development strategies for prophylactic HPV vaccines have been explored [17-20]. A potential vaccine candidate for the production of such prophylactic vaccines is the HPV-16 L1 major capsid protein which can self-assemble into VLPs. In fact, VLPs have been able to induce long lasting protective immune responses, and a human systemic vaccine based on highly purified VLPs (derived from different types of HPV: 6, 11, 16 and 18) is currently commercialized [22]. This vaccine could in theory prevent ~70% of CxCa cases [22,23]. Unfortunately, the high costs associated with its production and distribution hinders its widespread application. A lower-cost alternative could be the use of LAB for VLPs production instead. In fact, recombinant LAB could be used either as a cell factory for VLP production or as a mucosal vaccine. Indeed, mucosal vaccines are more suited and convenient than systemic vaccines, because they are easier to administer and relatively inexpensive to manufacture. In addition, since mucosal surfaces are the primary site of interaction between an organism and its environment and represent the major portal of entry for pathogens (*e.g*. HPV-16), development of effective mucosal vaccines is advantageous.

In this work, we used the model LAB, *L. lactis*, to produce HPV-16 L1 protein and we demonstrated by Western blot analysis that full-length L1 protein could be efficiently expressed in *L. lactis*. We also demonstrated for the first time that *L. lactis*-derived L1 protein can self-assemble into structures morphologically similar to VLPs intracellularly by TEM experiments. Most important, these VLPs display conformational epitopes as confirmed by ELISA analysis. This is the first time that HPV-16 L1 protein is produced in *L. lactis *using the NICE system [32]. This system allows controlled gene expression by addition of nisin, an antimicrobial peptide used as a natural preservative in the food industry, and more importantly, the level of gene expression can be up-regulated more than 1000-fold. Since, *L. lactis *is a non-colonizing bacterium, a system allowing the bacterium to be preloaded, such as with NICE system, with antigen before *in vivo *applications (vaccine development, for example) are highly desirable.

Note that recombinant *L. lactis *provide a possible means for targeting L1 protein to humans. However, the use of genetically modified microorganisms raises legitimate concerns about their survival and propagation in the environment and about the dissemination of antibiotic selection markers (such plasmids used in this study) or other genetic modification to other microorganisms. Thus, before recombinant bacteria could be applicable to humans a biologic containment of recombinant microorganisms is necessary [33].

In conclusion, this study demonstrates the potential of the use of recombinant LAB for the production of HPV-16 VLPs and suggests the development of a new mucosal prophylactic HPV-16 vaccine.

## Methods

### Bacterial strains and plasmids used

The bacterial strains and plasmids used in this work are listed in Table 1. *Lactococcus lactis *strains were grown in M17 medium (Difco) supplemented with 0.5% glucose (GM17) at 30°C without agitation. *Escherichia coli *was grown in Luria-Bertani at 37°C with vigorous agitation. Plasmid constructions were first established in *E. coli *and then transferred to *L. lacti*s by electrotransformation [24]. Plasmids were selected by addition of antibiotics as follows: for *L. lactis*, chloramphenicol (10 μg/ml); *for E. coli*, ampicillin (100 μg/ml) and chloramphenicol (10 μg/ml). Isolation of plasmid DNA was performed by using a Mini-Scale purification system (QIAGEN S.A.). Lysozyme (10 mg/ml) was added prior to the lysis step and incubated for 30 min (37°C) to prepare the protoplasts. PCR (Cetus apparatus; Perkin Elmer, Norwalk, CT) was performed using Vent DNA polymerase (Promega), and DNA sequences were confirmed by sequencing (MWG-Biotech AG). Restriction and DNA-modifying enzymes were used according to the supplier's recommendations.

**Table 1:** Bacterial strains and plasmids used.

**Strains**	**Genotype**		**Reference**
*E. coli *TG1	*supE, hsd, Δ5, thi, Δ(lac-proAB), F'(traD36 proAB-lacZΔM15)*		[30]
*L. lactis *MG1363	Wild type, plasmid free		[29]
*L. lactis *NZ9000	MG1363 (*nisRK *genes into chromosome), plasmid free		[27]
**Plasmids**	**Replicon**	**Plasmid characteristics; cloned cassettes characteristics**	**Reference**
pcDNA3-L1	ColE1	Ap^r^, plasmid containing the HPV-16 L1 sequence	[31]
pCR:TOPO	ori pUC	Ap^r^	Invitrogen
pCR:TOPO:L1	ori pUC	Ap^r^, plasmid containing HPV-16 L1 sequence without ATG start codon	This work
pCYT:E7	pWV01	Cm^r^; gene, expressed from P_nisA _encodes E7	[10]
pSEC:E7	pWV01	Cm^r^; gene, expressed from P_nisA _encodes SP_Usp_-*E*7 precursor	[10]
pCYT:L1	pWV01	Cm^r^; gene, expressed from P_nisA _encodes native L1 protein	This work
pSEC:L1	pWV01	Cm^r^; the gene expressed from P_nisA _encodes SP_Usp_-*L*1 precursor	This work

### Cloning of *L1 *gene from HPV-16 in *L. lactis*

A 1,606-bp DNA fragment encoding HPV-16 L1 protein (without ATG start codon) was PCR amplified from the pcDNA3-L1 plasmid (Kindly provided by Dr. Willem J.G. Melchers, Table 1) using primers *Nsi*I-L1 HPV16 (5'-G***ATGCAT***CA CAACAGGTGACTTTTATTTACATC-3') for the coding strand and L1 HPV16 (5'-GTTACAG CTTACGTTTTTTGCGTTTAGCAG-3') for the complementary strand. The PCR product was cloned into pCR:TOPO (Invitrogen), resulting in pCR:TOPO:L1. *L1 *gene was then purified from *Nsi*I/SpeI-cut pCR:TOPO:L1 and cloned into purified backbones isolated from *NsiI*I-SpeI-cut pCYT:E7 and pSEC:E7 resulting in pCYT:L1 and pSEC:L1 (Table 1). In the resulting plasmid pSEC:L1 (Figure 1), *L1 *gene is fused in frame with a DNA fragment containing the ribosome binding site and the signal peptide of *usp45 *(SP_Usp45_), the gene encoding Usp45, the predominant *L. lactis*-secreted protein [24]. In pCYT:L1 (Figure 1), the fragment encoding SP_Usp45 _is absent. In both pCYT:L1 and pSEC:L1, expression of L1 is controlled by the P_nisA _promoter [26]. These plasmids were introduced into *L. lactis *strain carrying the regulatory genes *nisR *and *nisK *(Table 1) [27]. As a negative control, NZ9000 was transformed with an empty vector (pGK^-^).

### Inducible expression of L1, protein analysis and immunoblotting

For the induction of L1 expression from the nisin promoter, strains were grown to an optical density (OD_600_) = 0.6, followed by induction with 1 ng/ml of nisin (Sigma) for 1 h. Protein samples were prepared from 2 ml of *L. lactis *induced cultures. After centrifugation (5 min, 10,000 rpm), the cell pellet and supernatant were treated separately. To compare the amounts of secreted and cell-associated proteins, both cell and supernatant fractions were concentrated and sample concentration calculated as follows: the equivalent of 1 ml of 1 OD_600 _unit of the sample (either cell or supernatant) was concentrated in a 100 μl final volume as described below, and 10 μl was loaded for SDS-PAGE. Supernatants were filtered on 0.2 μm pore-size filters (low protein retention; Millisar NML Sartorius) and treated with 1 mM PMSF and 10 mM DTT, followed by the addition of 100 μl of TCA (100%) to precipitate proteins. Samples were incubated for 10 min on ice, and proteins were recovered from the pellets after centrifugation at 4°C for 10 min at 13,000 rpm. The resulting pellet was dissolved in 1/20 volume of 50 mM NaOH. The cell fraction was obtained by cell lysis in 70 μl of lysis buffer (25% sucrose, 1 mM EDTA, 50 mM Tris-HCl [pH 8.0], and 10 mg/ml lysozyme) complemented with 1 mM PMSF and 10 mM DTT. After 30 min of incubation at 37°C, cells were lysed with 30 μl of 20% SDS. Equal volumes of 2× loading buffer were added to all samples and 10 μl of each sample were analyzed by SDS-polyacrylamide (10 and 12%) gel electrophoresis. Western blotting on PVDF membranes (Millipore) was carried out using an HPV-16 L1-specific monoclonal antibody (0.25 μg/ml, CamVir 1, BD Pharmingen). Immunodetection was performed with protein G-horseradish peroxidase conjugate (Bio-Rad) at a dilution of 1:5000 and an enhanced chemiluminescence kit (Dupont-NEN) as recommended by the suppliers.

### Transmission electron microscopy (TEM)

*L. lactis *strains were induced as described above and fixed with 2% glutaraldehyde in 0.1 M of sodium cacodylate buffer (pH 7.2) for 1 hour at room temperature. Samples were then post-fixed with 1% osmium tetroxide containing 1.5% potassium cyanoferrate before gradually dehydrating in ethanol (30–100%) and embedding in Epon resin. Thin sections (70 nm) were collected onto 200 mesh copper grids and counterstained with lead citrate before examination with Zeiss EM902 TEM operated at 80 kV (Carl Zeiss-France, MIMA2 Microscopy Platform, UR1196, INRA, Jouy en Josas, France). Images were acquired with a charge-coupled device camera (Megaview III) and analysed with ITEM Software (Eloïse, France).

### HPV-16 VLP ELISA

ELISA analysis was performed as described previously [28] with some modifications. Briefly, *L. lactis*-derived VLPs and L1 protein were recovered as follows: 10 ml of induced-*L. lactis *cultures were pelleted and resuspended in 3 ml of either PBS pH 7.0 (assembled VLPs) or in carbonate buffer (50 mM sodium carbonate, 1 mM MgCl_2_, 0.01 M of dithiothreitol, pH 10.0) (disassembled VLPs). Cells were then lysed with 0.1 g of acid-washed glass beads (150–210 μm, Sigma). Cellular debris were eliminated by centrifugation and 200 μl of supernatant containing VLP's or L1 protein were applied on ninety-six well plates (Nunc Maxisorp) and incubated at 4°C for 8 h. Plates were washed twice with PBS plus 0.1% Tween-20 and blocked at room temperature for 1 h with 5% non-fat dry milk in PBS. After plate washing, 100 μl of either an anti-HPV16 L1 capsid antigen (HP16M-1521-5, Austral Biologicals) or anti-L1 (BD Pharmigen) diluted 1:50 in 1% non-fat dry milk in PBS were added. After 2 h of incubation at room temperature the plates were washed and 100 μl of a secondary anti-mouse IgG antibody (alkaline-phosphatase conjugated goat, Sigma-Aldrich, France) diluted 1:2500 in 1% non-fat dry milk in PBS was added and the plate incubated 1 h at 4°C. The plates were washed and the reaction developed by addition of 200 μl of *p*-nitrophenyl phosphate substrate (Sigma-Aldrich, France) dissolved at 10 mg/ml in sodium carbonate buffer. The plates were incubated at room temperature until yellow colour was developed (30–60 min). Finally the reaction was stopped by the addition of 50 μl of 2 M NaOH. The absorbance was immediately measured at 405 nm (A405 nm).

## Competing interests

The authors declare that they have no competing interests.

## Authors' contributions

NGCP and LGBH conceived and designed the study with the help of PL. LGBH constructed the plasmids and strains. NGCP performed most experiments NGCP, LGBH and PL drafted the manuscript. All authors have critiqued and approved the final manuscript.
